# Precise and Immediate Action against Predatory Conferences

**DOI:** 10.4274/balkanmedj.galenos.2020.2020.1.001

**Published:** 2019-12-20

**Authors:** Zafer Koçak

**Affiliations:** 1 Department of Radiation Oncology, Trakya University School of Medicine, Edirne, Turkey

There is a strong possibility that many of us receive unsolicited spam emails offering publication in predatory journals or presentation opportunities at predatory conferences. As a young researcher, one can imagine the prestige of being invited to speak at an international conference. The question, “Does it look too good to be true?” needs to be asked. In recent years, it would not be surprising for such a scenario to happen to you.

In interesting research, conducted by Mercier et al. (1), an investigation was conducted into all unsolicited electronic invitations received from predatory publishers or fraudulent conferences, following a junior academician’s first publication. Over a period of 12 months, 210 of the 502 invitations were about attending, speaking at, or organizing the conference. These invitations came from 18 different organizers and the meeting places were Europe (46.2%), North America (31%), Asia (20.4%), or other continents (2.4%). Only 51 (24.2%) conferences were related to the author’s affiliations or research interests. The most recommended role was that of speaker (80%).

Does this threat to the integrity of scientific publication come only from these unsolicited emails? Are we not delaying the solution by merely blaming predatory publishers? Interestingly, a significant number of academics think that predatory conferences are not worth worrying about and proceedings publications are not important, compared with journal publications (2). As a result, researchers and institutions do not make much effort to solve the problem. Even the most famous universities in the developed world host predatory conferences (2).

In fact, this is not as new as we thought, as it was possible to see some traces of this threat to the integrity of scientific publication 15 years ago. Three MIT students signed off on a job that surprised the academic world. They developed the “SCIgen” program, which produces random nonsense computer science articles complemented by realistic looking graphics, numbers, and quotations. Their paper, called “Rooter: A Methodology for the Typical Unification of Access Points and Redundancy,” was accepted as a non-reviewed paper to the 9th World Multi-Conference on Systemics, Cybernetics and Informatics, in 2005 (3). Subsequently, a couple of years later, Jeffrey Beall coined the term “predatory meetings” as analogous to “predatory publications” and explained that the business model involves “conferences organized by revenue-seeking companies that want to exploit researchers' need to build their vitas with conference presentations and papers in published proceedings or affiliated journals.” According to Jeffrey Beall's blog, the number of predatory publishers increased from 18 in 2011, to 1155 in 2017 (4).

What is a predatory conference? Many of us are not experienced enough to identify these predatory conferences. The organizers of predatory conferences do not obey any ethical rules. They even hijack photos and biographies of eminent scientists from the internet. They use these names as bait to increase registration for conferences. Not only young academics but also experienced ones can fall into the trap of these organizers. Therefore, it is necessary to be aware of some common features pertaining to these predatory conferences. As described by McCrostie (2), to be classified as a predatory conference/organizer, three criteria are important: offering low-quality academic meetings for the primary aim of making money (not supporting scholarships); having no effective peer review process (allowing anyone to purchase a speaking slot); using deception (making false claims of peer review and hiding the true location of the company headquarters). There are two important guides/checklists that scientists can use to evaluate conferences. In addition to “Think. Check. Attend.” (5), which helps researchers and scholars to judge the legitimacy and academic credentials of conferences, Eaton’s checklist is one of the best guidelines for determining the legitimacy of a conference (Table 1) (6).

Is there a list of predatory conferences? I have been asked this question very often lately. Currently, there are two lists of international fake conferences which you can check on the internet (Table 2). In the Caltech list, over 90 organizers/conferences are listed under the heading of “Questionable Conferences” (7). In the Dolos list, 34 organizers/conferences are listed under the heading of “Dubious or Fake Conferences or Organizers” (8). However, in order to identify these predatory conferences, at the national level, it is helpful to follow the guidelines given above.

As stated in our recent editorial (9), Turkey is among the first five countries listed in the context of predatory publishing. Therefore, individual and institutional urgent measures are mandatory to raise awareness of this fraudulent publication practice. Under the new incentive allowance system, implemented in Turkey since 2016, researchers are paid not only for published papers but also for papers presented at any international conference. As can be seen in the literature, this system has some positive effects in some countries, but has had some undesirable consequences for others (10). Therefore, it is worth exploring the pros and cons of the new financial support policy for Turkish researchers.

This question was addressed in a study by Demir (11). He scanned the Roth’s list (Caltech library) to determine the number of Turkey-addressed papers in questionable conferences over the course of two years before (2014-2015) and after (2016-2017) the implementation of the ex-post funding system. He reported that the number of papers presented at questionable conferences increased surprisingly (49 in 2014--2015 vs 408 in 2016--2017) after the implementation of the ex-post funding system. He noted that this 732% increase in papers presented at questionable conferences could not be explained by an increase in the number of researchers.

As the number of such conferences and the number of participants increases day by day, some urgent and more effective measures should be taken by the academy. As an immediate action, publications or presentations at these predatory conferences, in developing countries, such as Turkey, should be removed completely from the scope of the incentive allowance system. The papers presented at these conferences should not be considered in the evaluation of academic promotion and assignment. Furthermore, institutions and universities should not host predatory conferences and take necessary measures against researchers who attend or organize such conferences.

## Figures and Tables

**Table 1:**
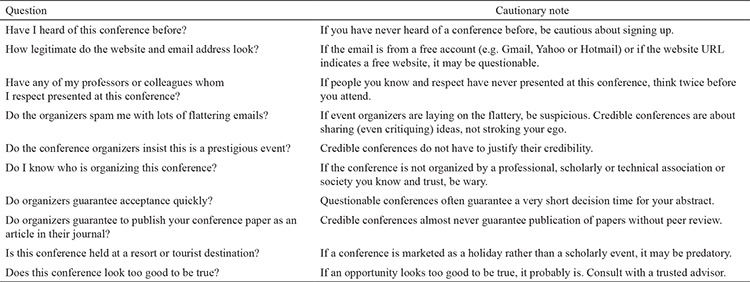
Eaton’s checklist to determine the legitimacy of a conference (6)

**Table 2:**
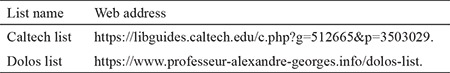
Websites for predatory (questionable or fake) conferences or organizers
